# The Beneficial Effect of Hydrogen on CO Oxidation over Au Catalysts. A Computational Study

**DOI:** 10.3390/molecules16119582

**Published:** 2011-11-16

**Authors:** Akhtar Hussain, Jose Gracia, J. W. Niemantsverdriet, B. E. Nieuwenhuys

**Affiliations:** 1 Physics Division, Pakistan Institute of Science and Technology (PINSTECH), P. O. Nilore, Islamabad 44000, Pakistan; 2 Schuit Institute of Catalysis, Eindhoven University of Technology, 5600 MB Eindhoven, The Netherlands; Email: Jose.Gracia@unizar.es (J.G.); j.w.niemantsverdriet@tue.nl (H.J.W.N.); 3 Leiden Institute of Chemistry, Leiden University, P.O. Box 9502, 2300 RA Leiden, The Netherlands;Email: nieuwe_b@chem.leidenuniv.nl (B.E.N.)

**Keywords:** PROX, DFT, gold, CO oxidation, adsorption

## Abstract

Density functional theory calculations have been carried out to explore the effect of hydrogen on the oxidation of CO in relation to the preferential oxidation of CO in the presence of excess hydrogen (PROX). A range of gold surfaces have been selected including the (100), stepped (310) surfaces and diatomic rows on the (100) surface. These diatomic rows on Au(100) are very efficient in H-H bond scission. O_2_ hydrogenation strongly enhances the surface-oxygen interaction and assists in scission of the O–O bond. The activation energy required to make the reaction intermediate hydroperoxy (OOH) from O_2_ and H is small. However, we postulate its presence on our Au models as the result of diffusion from oxide supports to the gold surfaces. The OOH on Au in turn opens many low energy cost channels to produce H_2_O and CO_2_. CO is selectively oxidized in a H_2_ atmosphere due to the more favorable reaction barriers while the formation of adsorbed hydroperoxy enhances the reaction rate.

## 1. Introduction

The oxidation of carbon monoxide on gold based catalysts has been studied extensively since Haruta *et al.* reported that Au nanoparticles supported on reducible metal oxides exhibit an extremely high CO oxidation activity [1]. This reaction is widely utilized as a model system because of its relative simplicity. Technologically, it has practical applications such as in exhaust gas emission control and purification of hydrogen for polymer electrolyte membrane fuel cells (PEMFCs).

Automotive exhaust gases contribute to atmospheric pollution and global warming [2,3,4]. PEMFCs are potentially an attractive and clean energy source for vehicle propulsion and auxiliary power units. Hydrogen storage and distribution can be avoided by producing hydrogen locally (on-board) from gasoline, methanol or natural gas via steam-reforming (e.g., CH_3_OH + H_2_O ➔ CO_2_ + 3H_2_) or partial oxidation (e.g., CH_3_OH + 1/2O_2_ ➔ CO_2_+ 2H_2_) combined with the water-gas shift reaction (CO + H_2_O ➔ CO_2_ + H_2_) [3,4,5]. Unfortunately, both steam reforming and partial oxidation produce a considerable amount of CO as a by-product. Traces of CO (>20 ppm) in the hydrogen gas deteriorate the performance of the Pt electrode of PEMFCs at the operating temperature, typically 60–100 °C [6]. The most promising approach to remove CO from H_2_ is by preferential oxidation of CO (PROX) [7,8]. Hence, an efficient PROX catalyst should have a high activity for CO oxidation (to make CO_2_) in the presence of a large excess of hydrogen and a low activity for H_2_ oxidation (water formation is undesired) at the operating temperatures of PEMFC [9]. However, these requirements are hard to meet because on most of the noble metal catalysts hydrogen oxidation is faster than CO oxidation [4,5].

For selective CO removal in an H_2_ stream, supported Au catalysts are potentially advantageous over other noble metal catalyst due to their extraordinary high activity for CO oxidation as well as their unique property that CO oxidation is faster than H_2_ oxidation in the relevant temperature range of the PEMFC [7,9,10]. The significant differences between Au and the platinum group metals (PGM) catalysts are the lower adsorption energies of CO and O on Au. At higher temperatures the CO coverage becomes small, allowing more hydrogen to dissociate and react with oxygen resulting in a decrease in the selectivity towards CO_2_. At low temperatures hydrogen adsorption is blocked by CO (T < 100 °C). On gold the selectivity towards CO_2_ is high in the temperature range relevant for fuel cell applications (~80 °C).

Interestingly, CO oxidation is enhanced by the presence of H_2_ in the feed under PROX conditions [7,11]. Different mechanisms have been proposed to explain the effect of H_2_ on CO oxidation. Grisel and Nieuwenhuys [7] observed a pronounced effect of H_2_O and H_2_ on the reaction rate for CO oxidation even at room temperature. The effect was ascribed to a beneficial role of surface OH groups in CO oxidation. The promotional effect of H_2_ on CO oxidation has been investigated on a Au/TiO_2_ catalyst by Piccolo *et al.* using infrared spectroscopy [11]. They suggested a mechanism that involves an OOH (hydroperoxy) intermediate. In another study a number of supported Au model catalysts viz. Au/Al_2_O_3_, Au/ZrO_2_ and Au/TiO_2_ were studied under H_2_ rich conditions [12]. H_2_ dissociation was proposed to occur on the Au particles. It was further suggested that CO is oxidized by a reaction between adsorbed CO and adsorbed OOH species (or any H_x_O_y_ species). According to another mechanism formate and carbonate species formed during the reaction over Au/TiO_2_ catalyst represent side products/inhibitors, but do not take part in the reaction as reaction intermediates. A H_2_-rich atmosphere was found to inhibit the formation of formates [13].

In the present computational study we have investigated CO oxidation in the presence of H_2_ on Au surfaces and addressed the issue how H_2_ can facilitate O_2_ dissociation and enhance CO oxidation. It has been established that the gold particle size plays a crucial role in determining the catalytic activity. For many reactions only gold nanoparticles smaller than ~5 nm contribute to the catalytic activity. These nanoparticles are characterized by a relatively high concentration of low-coordinated surface atoms. In an earlier paper [14] we reported on the pronounced effect of the coordination number of Au atoms to which CO and NO molecules bind. In the present study we have used various model systems in order to examine the effect of the nature of the site (structure and coordination of the surface atoms) on the activity: the densely packed (100), the stepped (310) surface and diatomic rows on the (100) surface. These diatomic rows exhibit sites may be present on Au-14 and Au-29 clusters which have been reported to be very efficient in H_2_ splitting [15].

## 2. Computational Details

We used the Vienna *ab-initio* simulation package (VASP) [16] which performs an iterative solution of the Kohn-Sham equations in a plane-wave basis set. Plane-waves with a kinetic energy below or equal to 400 eV were included in the calculations. The exchange-correlation energy was calculated within the generalized gradient approximation (GGA) proposed by Perdew and Wang (PW91) [17]. The electron-ion interactions for C, O, H and Au atoms were described by the projector-augmented wave (PAW) method developed by Blöchl [18]. This is essentially a scheme combining the accuracy of all-electron methods and the computational simplicity of the pseudo potential approach [19].

The relative positions of the Au metal atoms were initially fixed as those in the bulk, with an optimized lattice parameter of 4.18 Å (the experimental value is 4.08 Å) [20]. The optimized lattice parameter was calculated using the face-centred cubic (fcc) unit cell and its reciprocal space was sampled with a (15 × 15 × 15) k-point grid generated automatically using the Monkhorst-Pack method [21]. A first-order Methfessel-Paxton smearing-function with a width ≤0.1 eV was used to account for fractional occupancies [22]. Partial geometry optimizations were performed including the RMM-DIIS algorithm [23]. Geometry optimizations were stopped when all the forces were smaller than 0.05 eV/Å. Vibrational frequencies for transition states (TS) were calculated within the harmonic approximation. The adsorbate-surface coupling was neglected and only the Hessian matrix of the adsorbate was calculated [24]. The climbing-image nudged elastic band (cNEB) method [25] was used in this study to determine minimum-energy paths.

Closed shell CO, CO_2_ and H_2_ molecules were optimized at the Γ point by non-spin polarized calculations using a 10 × 10 × 10 Å^3^ cubic unit cell. Spin-polarized calculations in a 10 × 12 × 14 Å^3^ orthorhombic unit cell were performed for the open shell species H, OH, OOH and O_2_.

**Figure 1:**
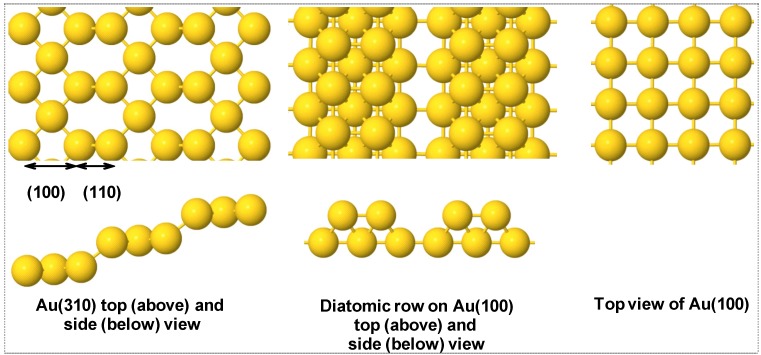
Top and side view of top layer of different Au surfaces used in this study.

A four layer slab model with the two top layers relaxed was chosen for creation of diatomic rows on Au (100). For this purpose, one row of Au atoms was removed from the surface as shown in Figure 1. The Au(100) and Au(310) surfaces were represented with a slab model using five-metal layers of which the top 2 relaxed for (100) and 11 layers with the top 4 relaxed for (310) [14] with a vacuum gap of >10 Å for both surfaces to separate the periodic slabs. For the Au(100) slab, we used a p(2 × 2) unit cell with the reciprocal space sampled with (5 × 5 × 1) k-point meshes. For the Au(310) p(2 × 1) unit cell (3 × 9 × 1) k-point meshes were used for sampling the reciprocal space.

## 3. Results

### 3.1. H_2_ Adsorption and Dissociation

Both molecular and dissociated hydrogen have been considered for their possible role in CO oxidation. In this section H_2_ adsorption and dissociation are reported for Au(100), the stepped Au(310) surface and for diatomic rows on Au(100). We have examined the adsorption of H_2_, both with the H-H molecular axis parallel and perpendicular to the surface. Our main conclusion is that H_2_ adsorption is independent of the surface structure, adsorption mode and adsorption site. The adsorption energy is only −0.02 eV, indicating a very weak interaction and, consequently, the metal to H_2_ distance is large keeping the H-H bond distance essentially equal to that of free H_2_ (0.75 Å).

H-atoms are stable on all the considered surfaces with varying strength. The highest adsorption energy, namely −2.41 eV, is found on the bridge position of diatomic rows. Adsorption on the nearby hollow position is only −1.88 eV. The adsorption energy of the H-atom decreases to −2.24 and −2.18 eV for bridge locations on the (100) and the (310) surfaces, respectively. H is about 0.3 eV less stable on hollow and top locations. For further details see Table 1.

**Table 1:** Adsorption energy of different species on Au(310)-p(2 × 1).

Species	Position	E_ads_ (eV)
H	(100)bridge at outer step	−2.18
	(100)bridge at inside step	−2.14
	bridge at step	−1.96
	(100) hollow	−1.9
	top (100)	−1.89
	top at step (110)	−1.98
O_2_ + H	bridge at step + (100)	−2.21
	bridge at inside step	
OOH	bridge at step	−1.00
OH + O	bridge at step + (100) hollow	−4.97
OOH + CO	bridge at step + (100) bridge at inside step	−1.31
OH + CO_2_	bridge at step + (100) bridge at outer step (across)	−2.2
O_2_ + H_2_	bridge at step	−0.19
OH+ OH	bridge at step + (100) hollow	−4.00
O + H	bridge at step + (100) bridge at inside step-	−5.49
OH	bridge at step	−2.31
OH + H	bridge at step + (100) bridge at inside step	−4.44
H_2_O	top at step (flat)	−0.23
H_2_O + O	bridge at step + (100) bridge at outer step	−3.46
CO + O	top at step + (100) hollow	−3.61

Coadsorbed H-atoms have an adsorption energy per hydrogen atom of −2.38, −2.18 and −2.04 eV on diatomic rows, (100) and (310), respectively, demonstrating the absence of any significant effect of lateral interactions between the hydrogen atoms on all the systems studied.

In the next step, dissociation of H_2_ was studied. These calculations are also of interest for the reverse reaction, recombination of 2 H-atoms. On (100), the four fold hollow site with H_2_ having the axis horizontal and thus parallel to the surface was taken as the initial state and two hydrogen atoms sitting on bridge sites were taken as the final configuration. The final state is energetically higher suggesting an endothermic reaction with a reaction energy of +0.21 eV. The dissociation barrier was calculated to be 0.96 eV. The transition state was found to be symmetric and the hydrogen atoms were 1.04 Å far apart in the transition state. A similar energy barrier of 0.98 eV but with a comparatively more endothermic (0.5 eV) reaction was observed on the terrace of (310), see Table 2. Therefore, the reverse reaction which is important on gold surfaces, e.g., for the water gas shift reaction, is both energetically and thermodynamically more favorable on (310) than on (100). In the transition state the H atoms are 1.45 Å separated.

**Table 2:** Activation barrier, reaction energy and H-H bond length and imaginary frequency in TS on different gold surfaces.

Surface	Initial position	Ea (eV)	ΔE (eV)	dH-H (Å)	ν (cm^−1^)
Diatomic rows Au(100)	Top	0.69	−0.16	1.41	1044*i*
Au(100)	hollow	0.96	0.21	1.04	241*i*
Au(310)	(100) top	0.98	0.50	1.45	322*i*

**Figure 2:**
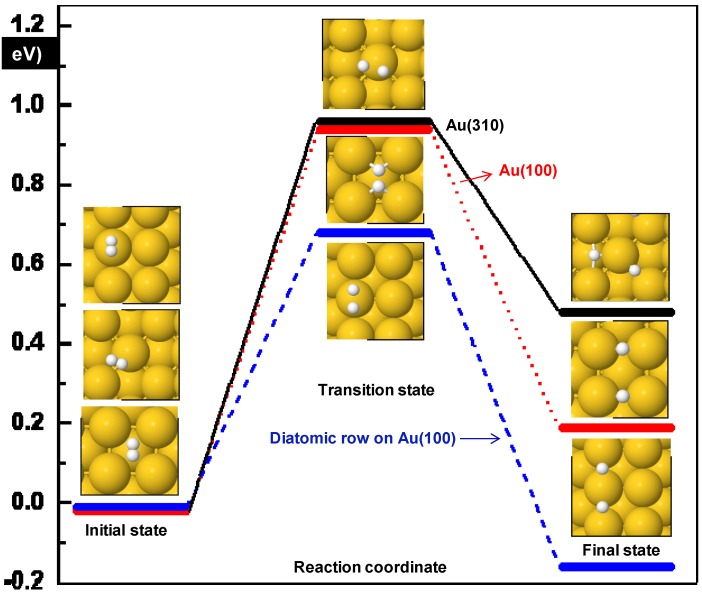
Overview of the results for H_2_ dissociation. Diatomic rows on Au(100) surface offer significantly lower activation barriers.

Although diatomic rows on (100) surface do not help to adsorb H_2,_ they may play an important role in direct dissociation of H_2_. The activation barrier as shown in Figure 2 and Table 2 is only 0.69 eV. This relatively low barrier suggests that H_2_ may split on this system below room temperature. The previously discussed surfaces were favorable for H_2_ formation but this surface with diatomic rows is thermodynamically favorable for H_2_ dissociation. The product state with two H-atoms on bridge locations is stabilized relative to the H_2_ initial state by −0.16 eV. In the transition state two participating hydrogen atoms are at 1.41 Å away and its interaction has an imaginary frequency of 1044 cm^−1^.

Other computational studies reported concerning the interaction of hydrogen with Au are in agreement with our results [5,15,26,28,29]. For instance, an E_a_ of 1.13 eV to dissociate H_2_ on Au(111) surface is reported by Oetjen *et al.* [5].^.^ Barrio *et al.* employed a PW91 functional to examine H_2_ adsorption and dissociation on the Au (100) and (111) surfaces and on Au_14_ and Au_29_ clusters [15]. They found that the flat surfaces are not active (E_ads_ −0.02 eV) towards H_2_ adsorption. However, spontaneous dissociation was reported on clusters with stabilization energy of −0.30 to −0.40 eV. A recent DFT study [28] of adsorption and dissociation of molecular hydrogen on the Au(111) and Au(001) surfaces, mono-atomic rows in an extended line defect and different Au nanoparticles reveals that low coordinated Au atoms are required for H_2_ dissociation. These low coordinated Au atoms may be present on nanoparticles or at extended line defects. However, the (111) and (100) single crystal surfaces are not active towards H_2_ dissociation in agreement with our study. Our diatomic row model contains an ensemble of four low-coordinated atoms, and the activation barrier is reduced considerably.

Experimental studies also support the model that molecular hydrogen is not adsorbed on clean extended surfaces of gold, and that hydrogen dissociation only occurs on thin non-sintered gold films [29] or supported gold nanoparticles [30,31,32]. Therefore, it has been proposed that low coordinated gold atoms are required for dissociative adsorption of hydrogen. These atoms are present on, for example, the corners or edges of gold nanoparticles [28,29].

#### 3.1.1. Hydroperoxy Formation and Decomposition on Au (O_2_ + H ➔ OOH ➔ OH + O)

Formation of adsorbed hydroperoxy has been identified in recent experimental studies concerning the PROX reaction [11,33,34]. We considered its formation from the interaction of molecular oxygen and predissociated hydrogen atoms present on the stepped (310), the diatomic rows on (100) and the (100) surfaces. Later in this paper, the interaction of molecular hydrogen with molecular oxygen will be discussed.

**Figure 3:**
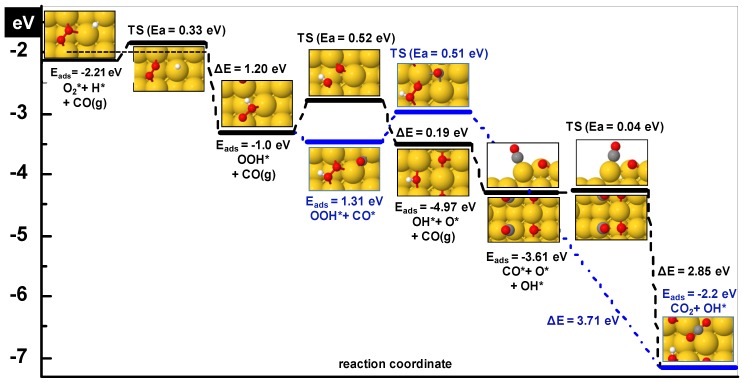
Reaction profile for CO_2_ formation in the presence of H on Au(310). After OOH formation, two paths have been explored: a) decomposition of OOH into O and OH where the formed O subsequently reacts with CO to form CO_2_ and b) via direct reaction of CO with OOH. Activation energy and reaction energy are indicated for each elementary step. Zero level corresponds to gas phase CO, O_2_, H and the clean slab. The horizontal dashed line indicates the desorption level of O_2_.

First, OOH formation and dissociation have been examined on the stepped (310) surface. Co-adsorption of H and O_2_ was investigated considering a few sets of configurations but here, only the most stable one shown in Figure 3 is described because the energy differences are trivial. O_2_ is placed on its favorite adsorption configuration with an adsorption energy of −0.17 eV, the bridge at the step, and H on the (100) bridge at the inside step. The adsorption energy of the coadsorbed system is −2.21 eV (the sum of the adsorption energies in the separate unit cells would be −2.31 eV) which indicates that the coadsorbed species slightly repel each other.

Adsorption of the hydroperoxy (OOH) intermediate was examined for a few locations by adding an H atom to one of the O-atoms of the O_2_ molecule. The most stable adsorption was found for the configuration shown in Figure 3. Its adsorption energy on this site is −1.0 eV. It is important to note here that addition of H to the O_2_ molecule results in a significant increase of the Au-(O-O) interaction. During the optimization procedure, the O_2_ molecule that is pushed towards the (100) bridge at the outer step becomes activated and the O-O bond length increases markedly from 1.33 to 1.47Å. Consequently, the O-O bond becomes weaker, which is important for O_2_ dissociation. In the adsorbed state the O atom of O_2_ near the gold atom forming the step is at a distance of 2.13 Å and the second O atom is 2.52 Å away from the gold atom on the terrace. The O-H bond length is 0.99 Å. The hydroperoxy species is energetically 1.2 eV more stable than its coadsorbed constituents O_2_ and H, and, hence, its formation is thermodynamically favorable. 

The transition state for H insertion has an activation barrier of 0.33 eV and is followed by an exothermic (−1.2 eV) reaction, see Table 3. In the transition state the H atom has moved from the (100) bridge at the inside step to the (100) top at the terrace, and the O-O bond length shortens to 1.31 Å. The O_2_ molecule slightly shifts in the direction opposite to the approaching way of H. The O-H bond separation is 2.53 Å. The transition state looks like the initial one because the reactants remain close to their initial positions.

**Table 3:** Activation barrier, reaction energy and bond length and imaginary frequency in TS on Au(310)-p(2 × 1).

Step	Ea (eV)	ΔE (eV)	d_TS_ (Å)	ν (cm^−1^)
O_2_* + H* ➔ OOH*	0.33	−1.2	2.53	112i
OOH* ➔ OH* + O*	0.52	−0.21	2.07	388i
CO* + OOH* ➔ CO_2_* + OH*	0.50	−3.71	-	
O_2_* + H_2_* ➔ OH* + OH*	1.95	−2.83	1.46	
OH* + OH* ➔ H_2_O* + O*	0.1	−0.06	1.85	
O* + H* ➔ OH*	0.19	−1.62	1.87	533i
OH* + H* ➔ H_2_O*	0.23	−1.1	1.86	277i
CO* + O* ➔ CO_2_	0.01	−2.85	3.29	53i
**Overall Reaction**				
CO + O_2_ + H_2_ ➔ CO_2_ + H_2_O				

To discern the O-OH bond scission barrier, co-adsorption of O and OH has been studied on different locations. For the preferred case on the (310) surface OH occupies its most favored position on the bridge at the step while O is on a four fold (100) hollow (Figure 3) on the terrace. An adsorption energy of −4.97 eV for the co-adsorbed system was found with respect to gas phase values of OH and atomic O; this is ~0.40 eV less than with the adsorbates at infinite separation. This shows the presence of repulsive interactions and suggests that the adsorbates will move further away to relieve the repulsion. Adsorbed OH (bond length 0.98 Å) is tilted with the H atom pointing towards the bottom of the step as shown in Figure 3 and the O-atom of OH is at a distance of 2.38 and 2.29 Å from the nearest gold atoms forming the step. The dissociated O-atom occupies a position slightly pushed away from its symmetrical position at (100) hollow towards the bottom of the step. An activation barrier of 0.50 eV is required to break the O-O bond and the reaction products are stabilized by 0.21 eV suggesting a slightly exothermic reaction. In the transition state OH remains tilted and is bonded to the top position at the step and O is bridge-bonded at the (100) outer step. It is worth mentioning here that the barrier to dissociate O_2_ with the assistance of hydrogen has been reduced to less than one half of that found in the absence of hydrogen on the (310) surface. This is an important result and in line with the experimentally found beneficial effect of H_2_ on CO oxidation, in relation to PROX. The O produced can easily be removed by CO to form CO_2_ and OH can be reduced to produce water or can react with another OH to provide water and O.

**Figure 4:**
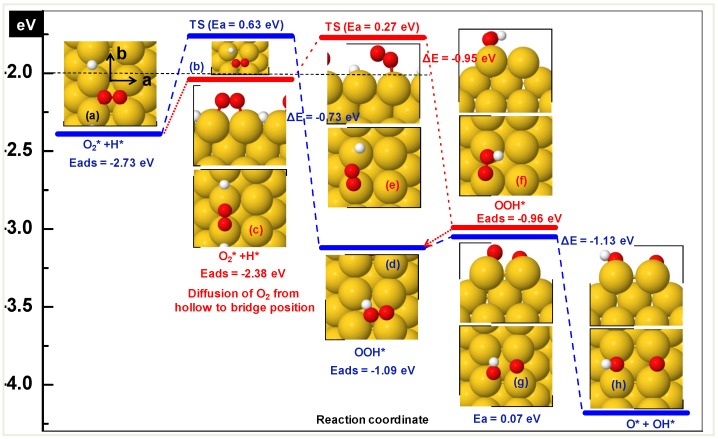
Potential energy diagram for hydroperoxy formation and decomposition on diatomic rows on the Au (100) surface. The horizontal dashed line shows the desorption level of O_2_. Zero energy corresponds to gas phase O_2_, atomic hydrogen and the empty slab.

Several co-adsorbed (O_2_ and H) configurations were investigated on the diatomic row on Au(100). Adsorption energies fall between −2.12 and −2.73 eV, see Table 4 where reference is gas phase O_2_ and H. The preferred configuration consists of O_2_ on the hollow and H on the bridge site (Figure 4a). For details concerning the adsorption energy of oxygen on this surface we refer to our previous paper [35]. The co-adsorbed system exhibits 0.30 eV repulsion with respect to the adsorbates at infinite separation.

**Table 4:** Adsorption energy of different species on diatomic row on Au(100)-p(3 × 2).

Species	Position	E_ads_ (eV)
H	bridge-b	−2.41
	hollow	−1.88
H + H	bridge-b + bridge-b	−4.75
	bridge-b + bridge-b inline	−4.72
O	bridge-b	−3.49
	hollow	−3.42
	3-fold hollow	−3.35
OH	bridge-b	−2.63
O_2_ + H	hollow-a + bridge-b	−2.73
	hollow-b + bridge-b	−2.43
	bridge-b + bridge-b	−2.38
	hollow-a + hollow	−2.12
OOH	hollow-a	−1.09
	bridge-b	−0.96
	hollow-b	−0.95
OH + O	bridge-b + bridge-b (H facing O)	−6.24
	bridge-b + bridge-b (O facing O)	−5.98
	bridge-b + bridge-a	−5.46
CO + OOH	bridge-b + bridge-b	−1.65
	bridge-b + bridge-x	−1.64
	hollow + top	−1.58
	hollow + bridge-b	−1.09
OC-OOH	top	stabilized by −0.73 eV
		w. r. t.
CO_2_ + OH	gas phase + bridge-b	most stable CO + OOH
		−2.74

The preferred configuration of hydroperoxy is with the O-O axis perpendicular to the row at the hollow site (E_ads_ = −1.09 eV with respect to gas phase OOH; Figure 4d). The bridged bonded OOH as shown in Figure 4f is 0.13 eV less stable relative to the preferred arrangement. The OOH formation for these two configurations has been studied starting from the co-adsorption arrangements of O_2_ and H shown in Figure 4a and Figure 4c. The minimum energy path (blue) corresponding to the most stable co-adsorption configuration, requires a barrier of 0.63 eV to form OOH. In the transition state (Figure 4b) the hydrogen atom moves from the bridge to top pushing nearby O-atom slightly backward and O-H distance is 2.1 Å. The product state is stabilized by −0.73 eV relative to the co-adsorbed configuration. The transition state is characterized by a unique imaginary frequency of 468 cm^−1^.

As an alternative, we examined another route (red) from the situation depicted in Figure 4c. This configuration is less favorable by 0.35 eV compared to the most stable one discussed above. However, the activation barrier is smaller, 0.27 eV. The total activation energy (0.35 + 0.27 = 0.62) is similar in both cases. In the transition state H approaches the top configuration pushing O_2_ slightly backward and the O-atom near to the H moves up (see Figure 4e). The distance between O and H is 2.63 Å. An imaginary frequency of 287cm^−1^ was found confirming the transition state character of this complex.

The OOH decomposes readily into adsorbed hydroxyl and oxygen, which reacts spontaneously with CO and CO_2_ is produced. The adsorption energy of co-adsorbed OH + O is −5.98 eV on the positions shown in Figure 4h demonstrating a weak repulsive interaction, because the sum of adsorption energies of OH and O in separate unit cells is −6.12 eV. However, co-adsorbed OH and O exhibit attractive interactions if the H is facing (opposite to the direction shown in Figure 4h) towards O by 0.12 eV. The activation barrier for decomposition is negligibly small (0.07 eV) as predicted by NEB calculations. Hence, both OOH formation and decomposition are thermodynamically favorable processes as is illustrated in Figure 4.

**Figure 5:**
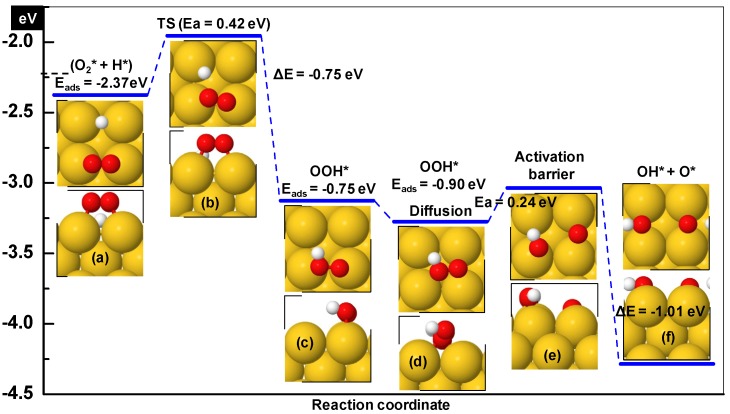
Potential energy diagram for hydroperoxy formation and decomposition on the Au(100) surface. The horizontal dashed line shows the desorption level of O_2_. Zero level corresponds to gas phase O_2_, atomic hydrogen and the clean slab.

Finally, for comparison and to determine whether an ensemble of Au atoms of higher coordination is able to decompose OOH, the process has been repeated on the (100) surface. Here the most stable configuration as shown in Figure 5a is less stable by 0.36 eV if compared with the diatomic rows on Au(100). However, the co-adsorbates neither repel nor attract each other. An activation barrier of 0.42 eV is needed for OOH formation, which is lower than that for the diatomic row structure. On the other hand, for OOH dissociation a slightly higher activation barrier of 0.24 eV is found, but O-O bond scission of OOH takes place on similar sites (see Figure 5e and Figure 4g). The OOH preferably occupies hollow positions on both surfaces. The E_ads_ of the OOH fragment decreases by 0.17 eV on (100) if compared to the diatomic row structure and a similar difference can be noted in the exothermicity towards the final products OH and O (see Figure 4 and Figure 5). The co-adsorption energy of OH and O in the configuration depicted in Figure 5f is −5.74 eV, which reflects a significant (0.53 eV) attractive interaction.

Activation energies for OOH formation (0.33, 0.62 and 0.42 eV) on the stepped (310), diatomic rows as well as on the (100) surfaces are small and the reaction is thermodynamically favorable. However, these barriers are competitive with desorption of O_2_. In each case the activation energy is slightly higher than the corresponding adsorption energies of O_2_ (−0.17, −0.52 and −0.12eV, see [35]). Although these differences are within the error limit of DFT, the possibility of this reaction to happen is limited. Practically, O_2_ is expected to desorb before OOH formation can take place on the studied surfaces. Once formed, further chemistry is very well possible, as decomposition of the OOH is energetically favorable on all the three surfaces. However, this decomposition is easier on the more compact (100) than on the stepped (310) surface. The lower barrier on Au(100) might be due to the nature of the site (hollow) constituted by four Au atoms on the (100) and on the diatomic row on (100) (see Figure 4 and Figure 5). Therefore, it is important to consider the role of the reducible metal oxide support/additive in the supply of OOH fragments.

#### 3.1.2. The role of a Reducible Metal Oxide for OOH Formation

Another possible route for formation of OOH on the Au surface is via spillover of OOH from the support to Au [36]. H_2_ can dissociate on Au. The adsorption energy of atomic hydrogen (−2.41 eV) on the diatomic rows on Au(100), is lower than that on TiO_2_(001) (−3.15 eV). This difference in adsorption energy may result in diffusion of H from Au to the support where O_2_ then may react with H to OOH [36]. After its formation the OOH may diffuse to the Au. On the clean TiO_2_ (001) surface the adsorption energy of OOH is −0.59 eV, [36] which is lower than on the Au surfaces (−0.96 to −1.09 eV). This provides a thermodynamic driving force for migration of OOH from the support to the gold.

#### 3.1.3. Reaction between Molecular Oxygen and Hydrogen (O_2_ + H_2_ ➔ OH + OH)

Is pre-dissociation of H_2_ on gold required for the formation of OH? We have explored the possibility of a direct bi-molecular reaction of H_2_ with O_2_ on Au(310). The very small adsorption energy (−0.19 eV) of co-adsorbed O_2_ and H_2_ reveals the minimal interaction between these molecules and of the molecules with the surface at the adsorbed positions. A high activation barrier of 2.05 eV is required for the direct reaction between the two molecules. The final state is highly stabilized with an exothermicity of −2.83 eV. As the molecules surmount the barrier, both molecules dissociate immediately forming two OH species. In the transition state taking place at the step, two H atoms are symmetrically 1.46 Å distant from the two O atoms and simultaneously the bond lengths of H_2_ and O_2_ increase from 0.75 to 0.95 Å and 1.33 to 1.54 Å, respectively. However, this reaction path is not energetically favorable and is unlikely to happen. The reactants will desorb before they have the chance to react with each other.

#### 3.1.4. Reaction between CO and OOH (CO + OOH ➔ CO_2_ + OH)

We have examined direct coupling of CO with the hydroperoxy intermediate as a possible route to CO oxidation. Co-adsorption of OOH and CO was investigated by keeping OOH near the bridge at the step and CO positioned on the (100) bridge at the inside step of Au(310) as shown in Figure 3 (blue path). In the co-adsorbed state the adsorption energy of the system is −1.31 eV with reference to gas phase CO and OOH. In this state the C atom of CO is 2.92 Å from the nearest O atom of OOH.

A moderate barrier of 0.50 eV is required for reaction of CO with OOH. In the transition state shown in Figure 3, the CO molecule has moved to a four fold (100) hollow site on the terrace and the OOH complex is slightly pushed away from the approaching CO. The carbon atom of CO is 2.71 Å away from the nearest O atom in the transition state. From this point, the optimization process does not yield a bicarbonate (HOOCO) complex; instead it dissociates immediately into OH and CO_2_, leaving OH bound to the gold atom at the step and CO_2_ at a large distance from the surface as shown in Figure 3. The dissociative adsorption of HOOCO is thermodynamically a highly exothermic process where the final state (OH + CO_2_) is 3.71 eV more stable in energy than coadsorbed CO + OOH. Hence, bicarbonate compounds are not formed on the surface. The results indicate that the O-O bond of O_2_ if weakened by interaction with H, immediately breaks as CO approaches the complex. Hence, bicarbonate compounds are not formed on the surface. This is another highly thermodynamically favorable channel which requires only 0.50 eV for CO oxidation, causing a rate enhancement. Here again, H plays an important role in the O-O bond scission for the subsequent CO oxidation reaction.

### 3.2. Hydrogen Peroxide Formation (HOOH)

Thus far we have discussed the effect of a single H atom attached to one O atom. To explore the effect of two H atoms attached to the two O atoms of the O_2_ molecule, we started by attaching another H atom to the already optimized structure of OOH. We were interested in the formation of O_2_H_2_. During the optimization process the O_2_ molecule dissociates into two hydroxyl groups (OHs) which move away from each other, lowering the system in energy towards a more stable state. As a consequence of this spontaneous process, in the final product state one OH group is bound in the tilted mode at an off top position to the gold atom forming the step, with the H atom pointing towards the upper terrace. The other OH group binds almost symmetrically on the (100) bridge at the outer step with the O-H bond axis roughly perpendicular to the surface. In this way, two OH groups make a configuration favorable for their mutual reaction (we discuss this later) for the production of H_2_O and O. The two O atoms are 3.44 Å apart in the final state. The energy released during this exothermic process, calculated with respect to the gas phase values of the OH, is −3.89 eV. This route for dissociation of molecular oxygen is the cheapest one as it requires no energy, but it does not directly produce CO_2_ or O.

### 3.3. Water Formation on Au

#### 3.3.1. OH Formation (O + H ➔ OH)

**Figure 6:**
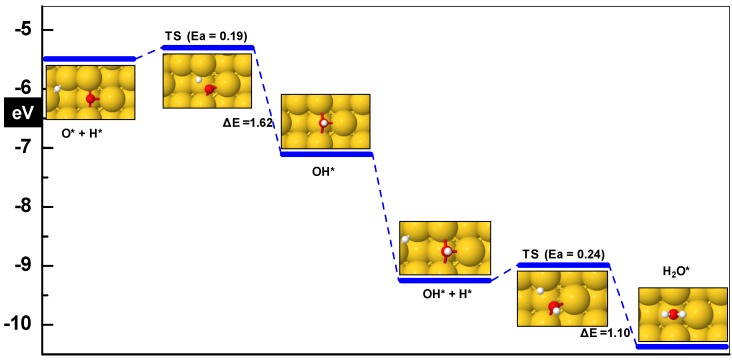
Water formation on the Au(310) surface from adsorbed O and H. Zero energy corresponds to gas phase O, H and the clean slab.

O-H bond breaking on the Au surfaces is quite difficult but on the other hand the reverse reaction is straightforward and thermodynamically favorable. A few coadsorption configurations were chosen to optimize. In the most stable state O is adsorbed on the bridge at the step and H occupies the (100) bridge at the inside step position (Figure 6). The adsorption energy of −5.49 eV is slightly higher (−0.03 eV) than for the case with these atoms adsorbed at infinite separations on these positions, thus showing a negligible attraction. This energy is −0.9 eV more negative than reported for Au(111) by Kandai *et al.* [9]. The product OH is 1.62 eV more stabilized than the O+H coadsorbed state making the reaction exothermic. In the transition state H is at a larger distance from its initial position and is bound to the Au atom at the step as shown in Figure 6.

The O-atom is slightly pushed towards the (100) bridge at the outer step, having a symmetrical 2.1 Å distance from the gold atoms forming this site and 1.87 Å apart from the approaching H. The transition state lies energetically 0.19 eV above its initial state with a unique imaginary frequency of 553 cm^−1^. A high barrier of 0.90 eV is reported for this step on the close packed Au(111) surface by Kandai et al [9] using GGA-PW91 exchange correlation functional.

#### 3.3.2. OH + H ➔ H_2_O

It has been established in the previous sections that the lower energy paths studied for PROX involving O_2_ activation produce OH species. Once H and OH species are present on the surface, they can react to produce water. For the most favorable case in the co-adsorption state, OH is bound to the bridge at the step having its O-H axis roughly perpendicular to the surface and the O-atom of OH sits symmetrically between the gold atoms forming the step; its distance from both near gold atoms is 2.46 Å. The H atom binds symmetrically at the (100) bridge at the inside step with a distance from the gold atoms equal to 1.76 Å. The co-adsorbed system has an adsorption energy of −4.44 eV calculated with respect to the gas phase values of OH and H. If compared with the adsorption energies of OH and H when these are at infinite separation, we do not find any attractive or repulsive interaction between the adsorbates. The reaction product water is 1.1 eV thermodynamically more stable than its reactants. A small activation barrier of 0.24 eV is needed to form water. On the other hand, dissociation of water on Au surfaces is very difficult. In the final product state water binds very weakly (−0.08 eV) at the step, but it can diffuse to the most stable (−0.23 eV) adsorption configuration on the top at the step. Water interacts very weakly with gold and may desorb at a temperature as low as 100 K. During the PROX reaction the presence of H_2_O has been observed recently by in situ infrared spectroscopy [11]. In the presence of O on the surface, CO will preferentially be oxidized to CO_2_ in comparison with H oxidation because of the lower barrier of 0.04 eV [36].

#### 3.3.3. Disproportionation of OHs (OH + OH ➔ H_2_O + O)

From the previous results, it is obvious that production of OH on the Au(310) by direct reaction of O_2_ with H_2_ is impossible. However, OOH decomposition into OH and O and/or reaction of H with OOH provides OH on the Au. These OH groups are reactive on Au and they either disproportionate into H_2_O and an O-atom or may react with CO to form COOH. The subsequent reaction for CO_2_ formation (from CO and O) follows almost spontaneously.

## 4. Discussion

A promotional effect of H_2_ on CO oxidation over supported Au catalysts has been observed in experimental studies [11,37]. However, this effect is not understood and, hence, an important issue in PROX is to investigate a feasible mechanism, which explains how H_2_ is involved and how it increases the CO oxidation rate. The presence of OH and OOH have been observed on Au catalysts under PXOX conditions in various investigations [11,33,34]. In an infrared spectroscopy study the presence of hydroxyl groups and hydroperoxy adsorbed on Au has been reported [28]. Based on these observations Piccolo *et al.* [28] have proposed that during PROX the formation of CO_2_ may result following the mechanism:

CO* + OOH* ➔ HCO_3_* ➔ CO_2_* + OH* ➔ CO_2_ + OH*

We have examined the formation of OH and OOH on the stepped (310) and diatomic rows model and (100) surfaces of gold. Based on our calculations and literature results, it can be concluded that H_2_ does not dissociate on low index and stepped surfaces but low coordinated Au atoms present on clusters can probably activate H_2_ readily [15,28,29,30]. We found that H_2_ dissociation on Au(100) with diatomic rows requires the relatively low energy barrier of 0.69 eV, suggesting that H-H bond breaking is possible below room temperature. Once H-atoms are present on the surface, hydroxyl and hydroperoxy species may be formed. Activation barriers are 0.33, 0.62 and 0.42 eV for the OOH formation and 0.50, 0.07 and 0.24 eV for decomposition into OH and O on the (310), diatomic rows and the (100) surfaces, respectively. Despite the small activation barriers, OOH formation is not likely on the basis of our chosen models because the respective adsorption energies of −0.17, −0.52 and −0.12 eV corresponding to above mentioned activation barriers are lower. An alternative and energetically feasible route for OOH on Au is its spillover from the support [36].

The OOH and OH on the Au surfaces are reactive species on Au catalysts and enhance the CO oxidation rate in various ways. For example: CO may react with OOH to form CO_2_ and OH by surpassing a moderate barrier of 0.50 eV in agreement with experimental studies [28]. This step is highly exothermic (−3.71 eV). Our calculations predict that OCOOH is not stable on the (310) surface which negates the formation of bicarbonates on the surface. OH groups may disproportionate into water and O-atoms, which are readily consumed by CO. OH and CO may also react to form COOH which may give CO_2_ and H or in turn react with another OH spontaneously to generate water and carbon dioxide as discussed in [38].

Hydrogen-peroxide formation was examined by adding H to OOH. However, during the optimization procedure the O-O bond scission takes place producing two OH groups on the (310) surface and releasing the energy equivalent of −3.89 eV.

The simultaneous presence of H, O, CO and OH species on the surface may lead to competition between CO and H oxidation. According to our model, CO oxidation is preferred over H oxidation due to a lower barrier and high exothermicity and the order of preference is CO oxidation (Ea 0.04 eV) > OHs disproportionation (Ea 0.10 eV) > H oxidation (Ea 0.2 and 0.24 eV). This observation is in line with the results of a DFT study performed on Au(111) surface by Mavrikakis *et al.* [9]. However, the concentration of the species on the surface may alter the situation and water is also formed as observed in a number of studies under PROX conditions which in turn also promotes CO oxidation [11,39,40,41,42,43,44,45].

Hence, introduction of H_2_ in a CO + O_2_ mixture opens several channels which cause O_2_ to hydrogenate and dissociate easily providing active O required for CO oxidation. The most important reactive intermediates are the hydroperoxy and OH species which in our opinion are responsible for the enhanced CO oxidation rate.

## 5. Conclusions

The mechanism and kinetics of CO oxidation in PROX over the Au surfaces have been investigated using DFT. The effect of H_2_ addition has been explored in detail. Our main conclusions are:

Direct bi-molecular reactions between O_2_ and H_2_ cannot take place because of the high activation barrier of 2.05 eV. Therefore, H_2_ dissociation is required for the PROX reaction. Au systems containing low coordinated Au atoms are capable to dissociate H_2_ below room temperature.

OOH formation on the (100), the diatomic rows on (100) and the (310) surfaces seems difficult because of the activation barriers of 0.42, 0.62 and 0.33 eV, respectively, on these surfaces which are slightly higher than the corresponding O_2_ adsorption energies of −0.12, −0.52 and −0.17 eV. Alternatively, hydroperoxy intermediates may migrate from the oxide support (For example TiO_2_) to Au particles. The OOH on the support is formed as a consequence of reaction of O_2_ with H (spilled over from Au).

The OOH on Au dissociates easily resulting in the formation of active oxygen needed for CO oxidation. Alternatively, it may react with CO giving OH and CO_2_ or it may react with another H to produce two OHs, which may disproportionate into H_2_O and O. For all these processes the activation barrier ranges from 0.0 to 0.50 eV. Consequently, O_2_ dissociation after hydrogenation occurs easily and the CO oxidation rate is enhanced. Formation of COOH and decomposition towards CO_2_ by reaction with OH is also a favorable process.

CO oxidation competes with H oxidation. On gold CO is oxidized preferentially as it requires a lower energy barrier (0.02–0.04 eV) than hydrogen oxidation (0.20 for OH and 0.24 eV for water formation). Hence, CO can selectively be oxidized in a hydrogen atmosphere.
